# Horse-Derived Ceramide Accentuates Glucosylceramide Synthase and Ceramide Synthase 3 by Activating PPARβ/δ and/or PPARγ to Stimulate Ceramide Synthesis

**DOI:** 10.3390/biomedicines11020548

**Published:** 2023-02-13

**Authors:** Tami Igarashi, Hiroki Yanagi, Masayuki Yagi, Masamitsu Ichihashi, Genji Imokawa

**Affiliations:** 1 Rosette Co., Ltd., Tokyo 140-0004, Japan; 2 Arts Ginza Clinic, Tokyo 105-0004, Japan; 3 Center for Bioscience Research and Education, Utsunomiya University, Utsunomiya 321-8505, Tochigi, Japan

**Keywords:** horse-derived ceramide, galactosylceramide, ceramide synthase 3, fatty acid elongase 4, glucosylceramide synthase, β-glucocerebrosidase, sphingomyelin synthase, reconstructed human epidermal equivalents, peroxisome proliferator activated receptors, keratinocytes

## Abstract

Horse-derived ceramide (HC), which contains galactosylceramides as its main component, significantly improves skin symptoms when applied topically to patients with atopic dermatitis. We speculated that efficacy resulted from the amelioration of epidermal ceramide metabolism, and we characterized those effects using reconstructed human epidermal equivalents. Lipid analysis, RT-PCR and Western blotting revealed that HC significantly increased the total ceramide content of the stratum corneum (SC), accompanied by significantly increased gene and/or protein expression levels of ceramide synthase (CERS) 3, fatty acid elongase (ELOVL) 4, glucosylceramide synthase (GCS), β-glucocerebrosidase, sphingomyelin synthase and acid sphingomyelinase. Mechanistic analyses using cultures of primary human keratinocytes revealed the marked stimulatory effects of HC on the mRNA expression levels of CERS3, ELOVL4 and GCS under high calcium-derived differentiation conditions. Signaling analyses demonstrated that an antagonist of PPARβ/δ significantly abrogated the HC-stimulated mRNA expression levels of GCS, CERS3 and ELOVL4. GW9662, an antagonist of PPARγ, significantly abolished the HC-up-regulated mRNA expression levels of GCS and ELOVL4, but not of CERS3. These findings suggest that HC has the distinct potential to accentuate the expression of GCS, CERS3 and ELOVL4 via the activation of PPARβ/δ and/or PPARγ to accelerate ceramide synthesis in the SC.

## 1. Introduction

Ceramides are a type of sphingolipid comprising a sphingoid base with amide linked to a saturated fatty acid, and they are major constituents of intercellular lipids in the stratum corneum (SC) of the skin. Ceramides play important roles in the water-holding and barrier functions of the SC [1,2]. Several dry skin diseases, such as senile xerosis [3], lamellar ichthyosis [4] and atopic dermatitis (AD) [5,6,7], have a ceramide deficiency, which is mechanistically attributable to the disrupted properties of the water-holding and permeability barrier functions involved in those skin diseases. Even in healthy human skin, ceramide levels in the SC have a general trend to decrease with increasing age [8], which contributes significantly to the appearance of age-related dry skin [5]. The close relationship between the ceramide deficiency and the impaired water-holding and barrier functions has been proven by abundant clinical data indicating that compensating for ceramides by utilizing synthetic pseudoceramides in the SC improves water-holding as well as barrier functions, resulting in ameliorating dry skin and enhanced skin sensitivity in senile xerosis and AD [9,10,11,12,13,14,15]. Based on the above evidence, although topical applications of natural ceramides or synthetic pseudoceramides are constitutively beneficial to dry skin symptoms, they have intrinsic difficulties in being widely used commercially due to their expensive cost and/or their requirement for high doses to exert their efficacy. Thus, it is intriguing to identify chemicals and/or plant extracts capable of stimulating ceramide synthesis in the epidermis, which could lead to increases of natural ceramides in the SC.

We previously reported that horse-derived ceramide (HC), which contains galactosylceramides (GalCer) as its main component, significantly improves skin symptoms by topical application at relatively low concentrations (<1.0%) for patients with mild-to-moderate AD, accompanied by a slightly increased water content in the SC [16]. Because GalCer cannot serve as a multilamellar-forming lipid as can natural ceramides or synthetic pseudoceramides to ameliorate the disrupted water-holding and barrier functions in the treated SC [10,11,12,13,14,15,17,18,19,20], we speculated that efficacy resulted from the amelioration of epidermal ceramide metabolism. GalCer is a glycosphingolipid with a lipophilic sphingosine and a hydrophilic galactose moiety attached via an ether linkage to sphingosine. GalCer is found abundantly in the myelin sheath of the central and peripheral nervous system. GalCer-deficient myelin loses its insulating properties and causes a severe dysmyelinosis that is incompatible with life [21]. GalCer was also reported to exist in pig skin and in cultured human keratinocytes [22,23]. As for the biological effects of GalCer on the skin or on keratinocytes, available evidence indicates that GalCer triggers the terminal differentiation of fetal rat skin keratinocytes in culture [24]. In mouse skin, topical application of GalCer increases β-glucocerebrosidase (GBA) activity in the epidermis, in concert with significantly increased ceramide levels in the epidermis as well as in the SC, while a similar up-regulated activity of GBA by GalCer treatment occurs in cultured normal human keratinocytes (NHKs) [25]. Recently, Kage et al. reported the effect of GalCer on SC intercellular lipid synthesis in reconstructed human epidermal equivalents (RHEEs) without modification when used just prior to the SC formation as described later [26]. However, that study failed to show any significant (*p* < 0.05) increase in levels of total ceramide or any ceramide species in homogenates of RHEEs and did not report any data of ceramide levels in the SC, which essentially regulates SC functions. There was no significant (*p* < 0.05) up-regulation of almost all ceramide synthesis-related genes examined, such as ceramide synthase (CERS) 3, glucosylceramide synthase (GCS), sphingomyelin synthase (SMS) 1, SMS2 and acid sphingomyelinase (ASM), except for GBA and serine palmitoyltransferase (SPT) 1.

Although the mechanistic association of the up-regulating effects of GalCer on GBA activity with an incremental effect on ceramide levels in the SC seems to be convincing, the biological mechanism involved remained obscure due to following reasons: GBA is a GlcCer-hydrolyzing enzyme that exists at a sufficient level within lamellar granules in the granular layers of healthy normal epidermis, reflecting there are no GlcCer residues in healthy normal SC. This indicates that unless its substrate GlcCer is up-regulated, increased levels of GBA proteins caused by GalCer treatment do not necessarily contribute to an increase in SC ceramide levels. Therefore, it was essential to determine the stimulatory effects on ceramide-metabolizing enzymes other than GBA.

Since ceramide synthesis occurs during the process of differentiation from the basal layer to the granular layer, it is difficult to precisely evaluate the level of synthesized ceramide that accumulates in the SC using monolayer primary cultures of NHKs. Therefore, we developed a new modified method using RHEEs in which the levels of ceramide in the SC can be evaluated after seven days of culturing starting just prior to the formation of the SC in the presence of several candidate chemicals to assess their stimulatory effects on ceramide synthesis [27,28,29,30]. The goal of this study was to characterize the accentuation of epidermal ceramide metabolism caused by treatment with HC using RHEEs and NHKs. Using RHEEs, we examined the effects of HC on ceramide levels in the SC as well as its influence on the expression of ceramide synthesis-related genes and proteins. Further, using NHKs, we analyzed the intracellular signaling mechanisms involved in the possible increased expression of ceramide synthesis-related genes and enzymes. This study shows for the first time that HC stimulates ceramide synthesis via the up-regulated expression levels of fatty acid elongases (ELOVL4), CERS3, GCS, GBA and SMS1, which results in significantly increased levels of total ceramides in the SC, which comprise significantly increased levels of Cer[EOS], Cer[NS/NDS], Cer[AS], Cer[NH] and Cer[AP]. Further analysis using NHKs indicated that the stimulatory effects on the gene expression levels of ELOVL4/CERS3/GCS and ELOVL4/GCS are mediated via the activation of peroxisome proliferator activated receptors (PPAR)β/δ and the PPARγ signaling pathway, respectively.

## 2. Results

### 2.1. Residual Levels of HC on RHEEs

**Figure 1:**
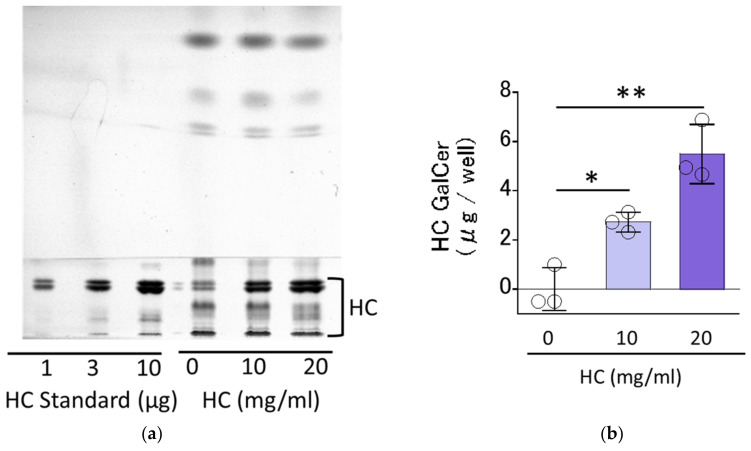
Residual levels of HC on RHEEs. (**a**) HPTLC chromatogram. (**b**) Levels of HC. A total of 4% HC glycerin aqueous solution (at 0, 10 and 20 mg/mL) was applied for 3 h on the surface of RHEEs, which were then washed with PBS. The RHEEs were then extracted with chloroform, methanol and PBS as described in the Section 4 Materials and Methods (Section 4.4 Evaluation of the residual level of HC applied on RHEEs) to obtain total lipids with applied and remaining HC. The total lipids were subjected to HPTLC analysis to measure the residual levels of HC in RHEEs. HC contents are expressed as µg/well. Bars represent mean ± standard deviation (SD), *n* = 3, Dunnett’s multiple comparison test. *: *p* < 0.05, **: *p* < 0.01.

We first determined the residual levels of HC on RHEEs after 3 h of treatment with a HC glycerin aqueous solution and its subsequent removal by washing with phosphate buffer saline (PBS). High-performance thin-layer chromatography (HPTLC) analysis of the extracted lipids from RHEEs revealed that distinct levels (2.72 ± 0.34 and 5.50 ± 0.98 µg/well, respectively) of HC remained in the RHEEs after the 3 h treatment with HC solution at 10 and 20 mg/mL and subsequent washing out by PBS (Figure 1).

### 2.2. Effect of HC on SC Ceramide Levels in RHEEs

**Figure 2:**
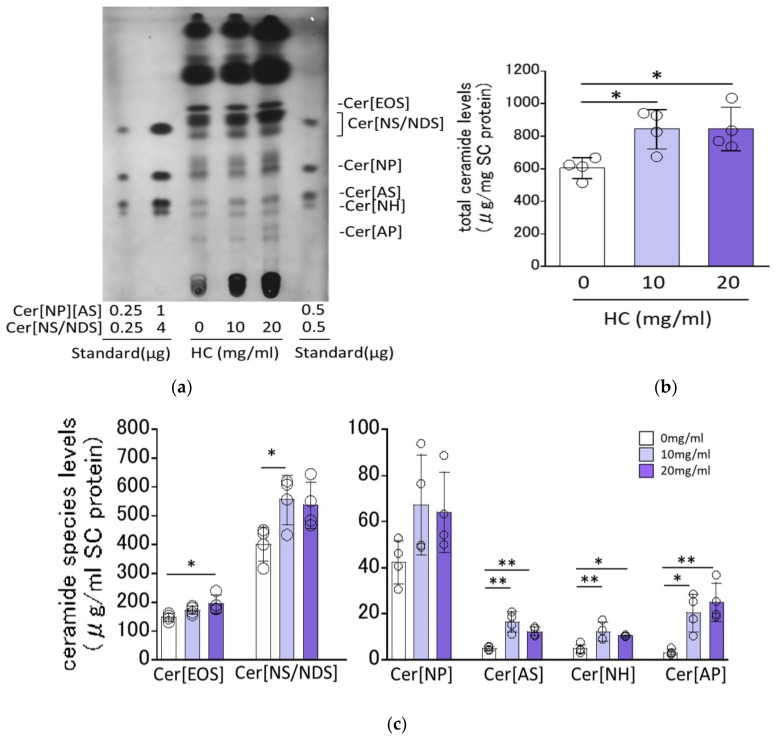
Effect of HC on SC ceramide levels in RHEEs. (**a**) HPTLC chromatogram. (**b**) Levels of total ceramides. (**c**) Levels of ceramide species. RHEEs were treated daily with HC (at 0, 10 and 20 mg/mL) for 10 days and the SC was separated from the epidermis after which total lipids were extracted as described in the Section 4 Materials and Methods. Total lipids extracted were subjected to HPTLC analysis. Ceramide contents are expressed as µg/mg SC protein. Bars represent mean ± SD, *n* = 3, Dunnett’s multiple comparison test. *: *p* < 0.05, **: *p* < 0.01.

The RHEEs were treated with HC at concentrations of 0, 10 and 20 mg/mL as described in the Section 4 Materials and Methods and were cultured for 10 days, after which they were treated with 0.05% trypsin to separate the SC from the epidermis. The isolated SC was subjected to HPTLC analysis to measure SC ceramide levels and their composition. HPTLC chromatograms of the extracted SC lipids demonstrated that various ceramide species, including Cer[EOS], Cer[NS/NDS], Cer[NP], Cer[EOH], Cer[AS], Cer[NH] and Cer[AP], were clearly detected and were assigned in accordance with previous studies [27,31] (Figure 2a). Quantitative HPTLC analysis demonstrated that total SC ceramides, expressed as µg/mg SC protein, were significantly increased at a HC concentration of 10 and 20 mg/mL compared with the control (0 mg/mL) (Figure 2b). Quantitative analysis of ceramide composition revealed that Cer[EOS], Cer[NS/NDS], Cer[AS], Cer[NH] and Cer[AP] were significantly increased by HC at 20, 10, 10 and 20, 10 and 20, and 10 and 20 mg/mL, respectively, while Cer[NP] was slightly but not significantly increased by HC at 10 and 20 mg/mL (Figure 2c).

As for the possibility that ceramides generated from HC by keratinocyte-derived galactocerebrosidase [32] act as an active lipid mediator to stimulate the observed ceramide synthesis, Kage et al. [26] demonstrated that when purified galactocylceramide was incubated with the homogenate of the RHEEs (layered human keratinocytes) at 37 °C, any free ceramides were not released, which suggests that there is no possibility of hydrolysis of HC applied to RHEEs to produce free ceramides which result in stimulating ceramide synthesis as reported by Ogretmen et al. [33].

### 2.3. Effect of HC on the Expression Levels of Ceramide Synthesis- and Keratinization-Related Genes in RHEEs

**Figure 3:**
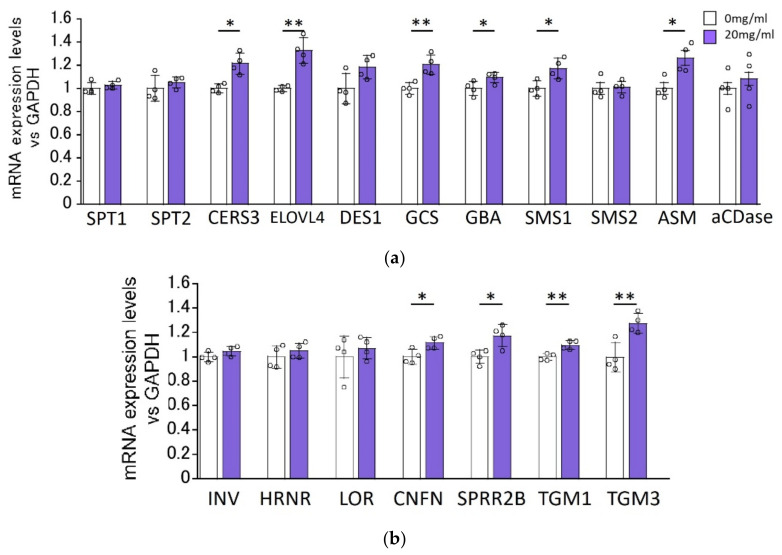
Effect of HC on the expression of ceramide synthesis- (**a**) and keratinization-related (**b**) genes in RHEEs. RHEEs were treated daily with HC (at 0 and 20 mg/mL) for 2 days and total RNA was extracted as described in the Section 4 Materials and Methods. mRNA expression levels were determined by real-time RT-PCR and are expressed as each mRNA level/glyceraldehyde-3-phosphate dehydrogenase (GAPDH). Bars represent mean ± SD, *n* = 3, Student’s *t*-test. *: *p* < 0.05, **: *p* < 0.01.

We determined the effects of HC on the expression levels of ceramide synthesis-related genes in RHEEs after 48 h of treatment. RT-PCR analysis revealed that mRNA levels of ceramide synthesis- and keratinization-related genes, such as ELOVL4, CERS3, GCS, GBA, SMS1, ASM, coniferin (CNFN), small proline-rich protein (SPRR)2B, transglutaminase (TGM)1 and TGM3, were significantly increased by treatment with 20 mg/mL HC, whereas mRNA levels of SPT1, SPT2, dihydroceramide desaturase (DES)1, SMS2, acid ceramidase (aCDase), involucrin (INV), hornerin (HRNR) and loricrin (LOR) remained unchanged by the same treatment with HC (Figure 3).

### 2.4. Effect of HC on the Expression Levels of Ceramide Synthesis- and Keratinization-Related Proteins in RHEEs

**Figure 4:**
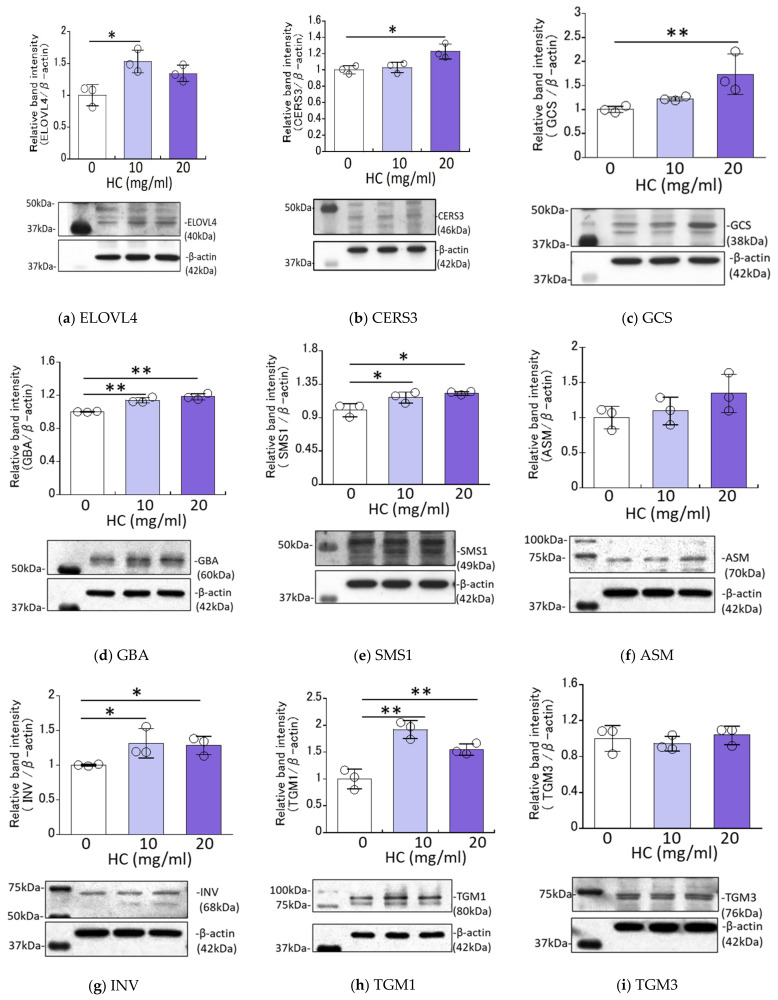
Effect of HC on expression levels of ceramide synthesis- and keratinization-related proteins in RHEEs. (**a**) ELOVL4, (**b**) CERS3, (**c**) GCS, (**d**) GBA, (**e**) SMS1, (**f**) ASM, (**g**) INV, (**h**) TGM1, (**i**) TGM3. RHEEs were treated with HC (at 0, 10 and 20 mg/mL) for 4 days and total proteins were extracted. Protein expression levels were determined by Western blotting as described in the Section 4 Materials and Methods and are expressed as each protein level/β-actin. Bars represent means ± SD, *n* = 3, Dunnett’s multiple comparison test. *: *p* < 0.05, **: *p* < 0.01.

We next determined the effects of HC on the expression levels of ceramide synthesis- and keratinization-related proteins in RHEEs. Western blotting analysis revealed that the protein expression levels of ELOVL4, CERS3, GCS, GBA, SMS1, INV and TGM1 were significantly up-regulated by treatment for 4 days with HC at 10 and/or 20 mg/mL (Figure 4). On the other hand, the protein expression levels of ASM and TGM3 remained unchanged by the same treatment (Figure 4).

### 2.5. Improvement of Skin Barrier Function of RHEEs by HC

**Figure 5:**
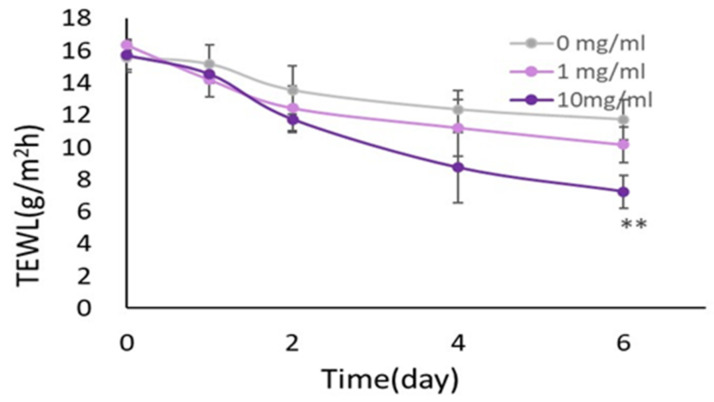
Effect of HC on the skin barrier function in RHEEs by TEWL analysis. RHEEs were treated with HC (at 0, 1 and 10 mg/mL) and were incubated for 6 days, and TEWL was measured at days 0, 1, 2, 4 and 6 of culturing as described in Section 4 Materials and Methods. During that period, the medium and samples were replaced every other day. Data represent the mean ± SD; *n* = 4. ** *p* < 0.01 vs. control.

We next determined the effects of HC on the skin barrier function on RHEEs. When HC suspended in PBS at 1 and 10 mg/mL was applied daily for 6 days on the surface of each RHEE, trans-epidermal water loss (TEWL) values measured on the surface of RHEEs exhibited significantly decreased values after treatment with HC at 10 mg/mL for 6 days in culture, compared to the non-HC-treated control (Figure 5).

### 2.6. Effect of HC on the Expression of Ceramide Synthesis- and Keratinization-Related Genes under Proliferating and Differentiating Conditions of NHKs

**Figure 6:**
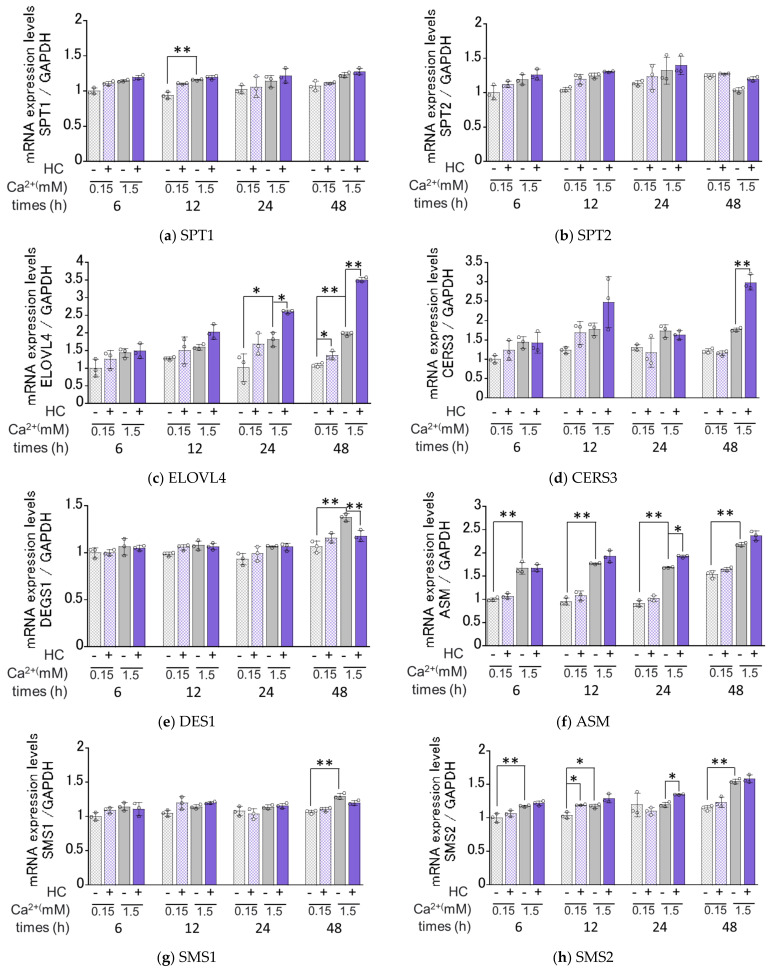

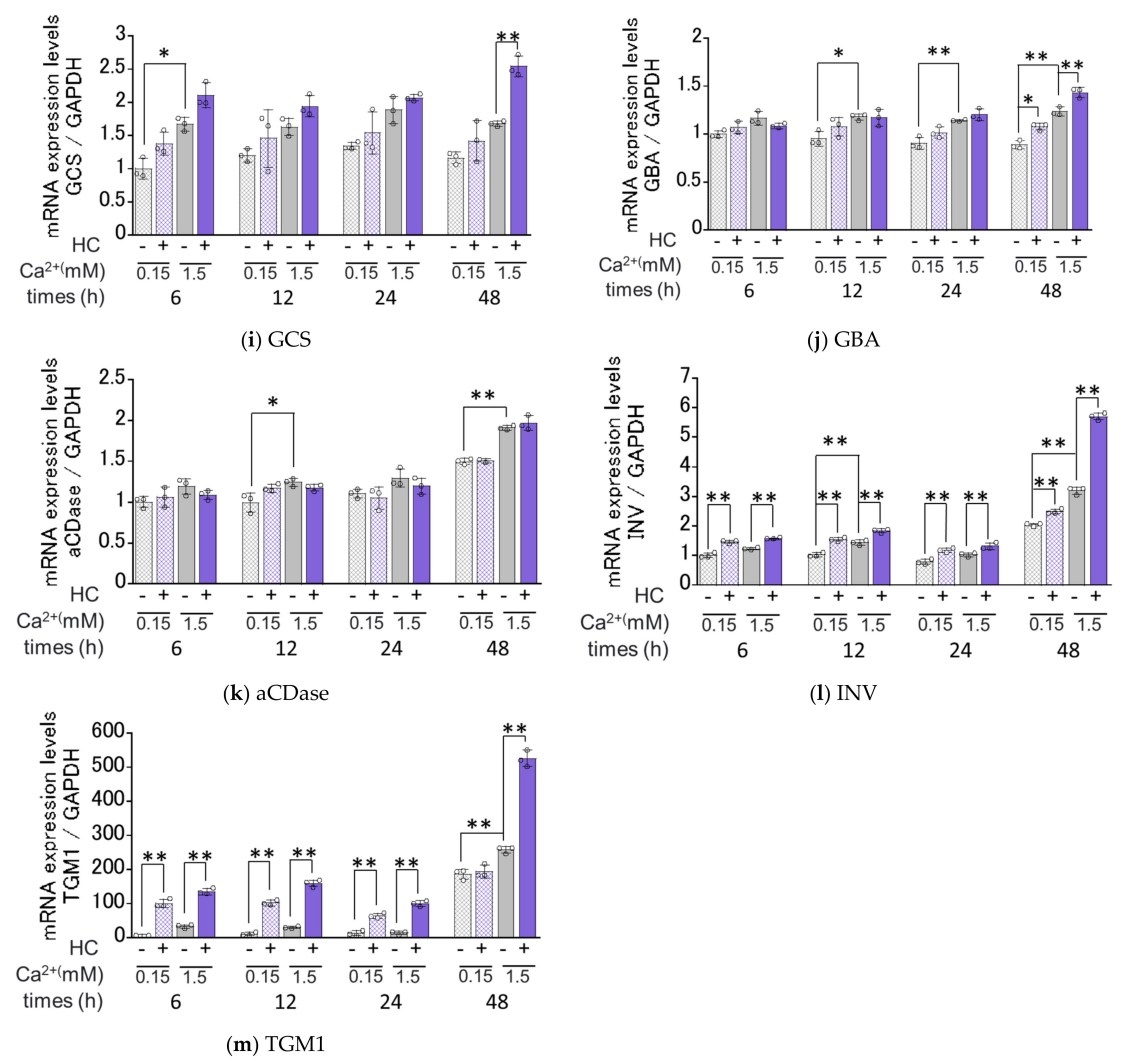
Effect of HC on the expression of ceramide synthesis- and keratinization-related genes in NHKs under proliferating and differentiating conditions. (**a**) SPT1, (**b**) SPT2, (**c**) ELOVL4, (**d**) CERS3, (**e**) DESG1, (**f**) ASM, (**g**) SMS1, (**h**) SMS2, (**i**) GCS, (**j**) GBA, (**k**) aCDase, (**l**) INV, (**m**) TGM1. NHKs were treated with HC (at 0 or 200 µg/mL) for 6, 12, 24 and 48 h in low-Ca^2+^ or high-Ca^2+^ conditions, after which total RNAs were extracted as described in the Section 4 Materials and Methods. mRNA expression levels were determined by real-time RT-PCR and are expressed as each mRNA level/GAPDH. Bars represent mean ± SD, *n* = 3, Tukey–Kramer multiple comparison test. *: *p* < 0.05, **: *p* < 0.01.

We next determined the effects of HC on the mRNA expression levels of ceramide synthesis- and keratinization-related genes under proliferating (0.15 mM Ca^2+^) and differentiating (1.5 mM Ca^2+^) conditions of NHKs. Real-time RT-PCR analysis revealed that in proliferating conditions, mRNA expression levels of SMS2, ELOVL4/GBA, INV and TGM1 were significantly increased by treatment with 200 µg/mL HC after 12 h, after 48 h, after 6/12/24/48 h and after 6/12/24 h, respectively, of culturing (Figure 6). In contrast, mRNA levels of SPT1, SPT2, CERS3, DES1, ASM, SMS1, GCS and aCDase remained unchanged by treatment with HC under the same proliferating conditions (Figure 6). In differentiating conditions, mRNA expression levels of ASM/SMS2, ELOVL4, CerS3/GCS/GBA and INV/TGM1 were significantly up-regulated by treatment with 200 µg/mL HC after 24 h, after 24/48 h, after 48 h and after 6/12/24/48 h, respectively, of culturing, while mRNA levels of SPT1, SPT2, SMS1 and aCDase remained unchanged. In contrast, the mRNA level of DES1 only was significantly down-regulated after 48 h of treatment with HC in the differentiating conditions of NHKs (Figure 6).

### 2.7. Effect of HC on the mRNA Expression Levels of PPARs under Proliferating and Differentiating Conditions of NHKs

**Figure 7:**
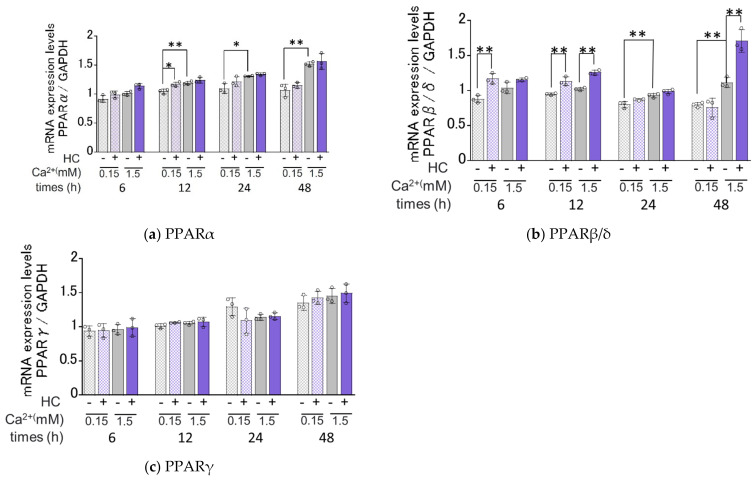
Effect of HC on the mRNA expression levels of PPARs under proliferating and differentiating conditions of NHKs. (**a**) PPARα, (**b**) PPAR β/δ, (**c**) PPARγ. NHKs were treated with HC (at 0 or 200 µg/mL) for 6, 12, 24 and 48 h in low-Ca^2+^ or high-Ca^2+^ conditions. Total RNAs were extracted as described in the Section 4 Materials and Methods. mRNA expression levels were determined by real-time RT-PCR and are expressed as each mRNA level/GAPDH. Bars represent mean ± SD, n = 3, Tukey–Kramer multiple comparison test. *: *p* < 0.05, **: *p* < 0.01.

Available evidence indicates that agonists of PPARs upregulate mRNA levels of ceramide synthesis- [34,35] and keratinization-related genes [36] and that the expression of PPARs at the gene and protein levels is enhanced by ceramide derivatives and their metabolites [37,38,39]. In order to elucidate the mechanisms involved in the HC up-regulated expression of ceramide synthesis- and keratinization-related genes, we determined its effects on the mRNA expression levels of PPARα, PPARβ/δ and PPARγ (Figure 7). Real-time RT-PCR analysis revealed that in proliferating conditions, mRNA expression levels of PPARα and PPARβ/δ, but not PPARγ, were significantly increased by treatment with HC at a concentration of 200 µg/mL after 12 h and 6/12 h, respectively, of culturing (Figure 7). In differentiating conditions, mRNA expression levels of PPARβ/δ were significantly up-regulated by the treatment with HC at a concentration of 200 µg/mL after 12/48 h of culturing, whereas mRNA expression levels of PPARα and PPARγ remained unchanged (Figure 7).

### 2.8. Effect of Agonists or Antagonists of PPARs on the HC-Stimulated Expression Levels of Ceramide Synthesis-Related Genes under Differentiating Conditions of NHKs

**Figure 8:**
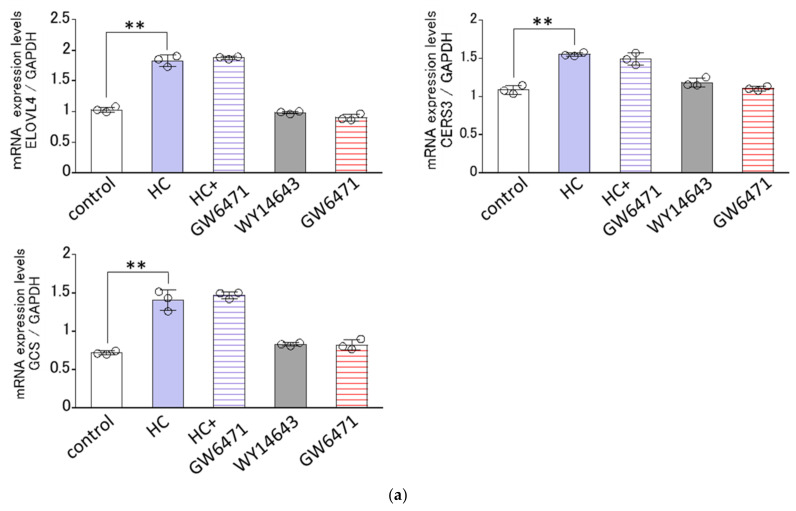

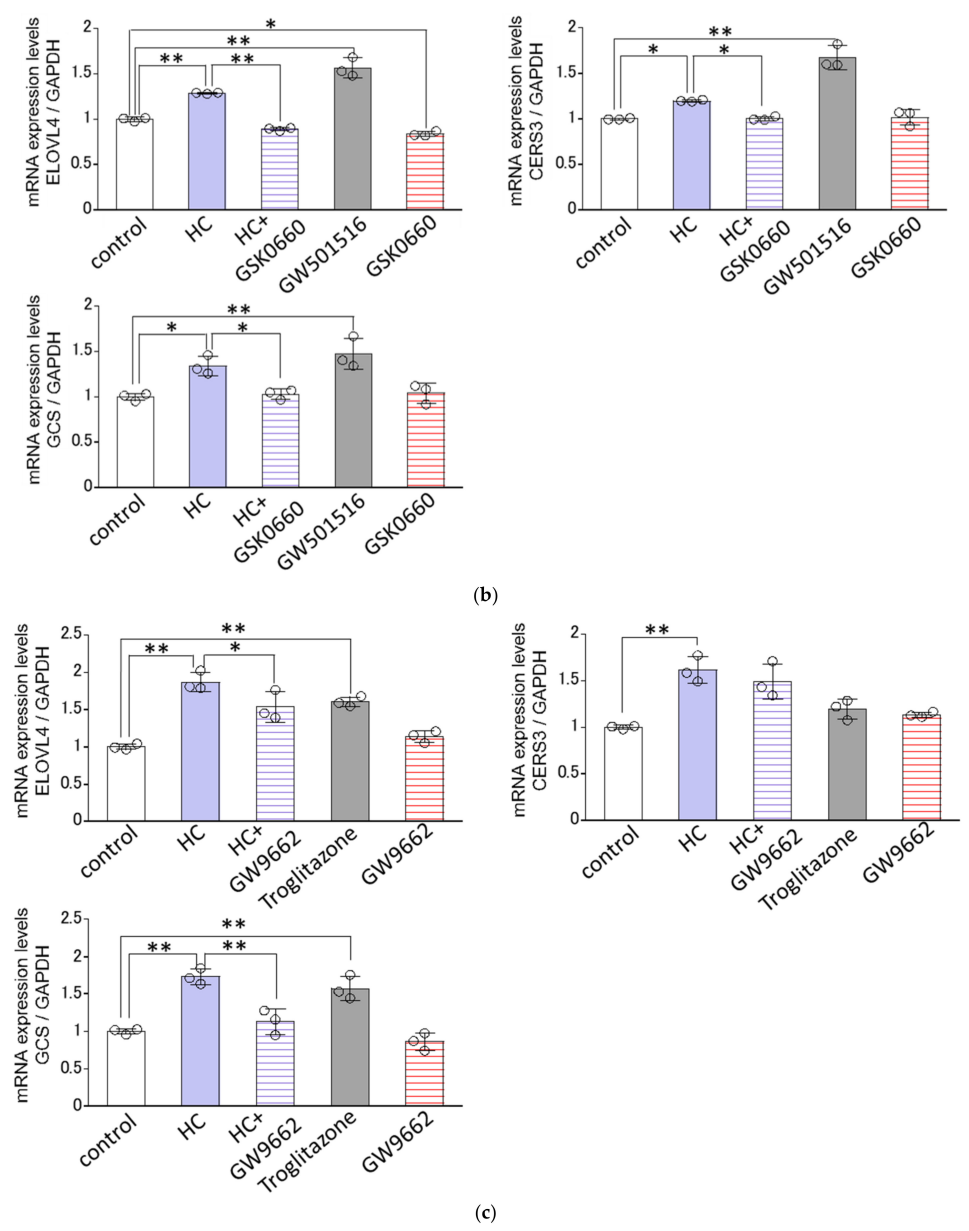
Effect of agonists or antagonists of PPARs on the HC-stimulated expression levels of ceramide synthesis-related genes under differentiating conditions of NHKs. Agonists/antagonists of (**a**) PPARα, (**b**) PPARβ/δ, (**c**) PPARγ. NHKs were treated with HC (at 0 or 200 µg/mL), with or without an agonist or antagonist of PPARs for 48 h in differentiating conditions and total RNAs were extracted as described in the Section 4 Materials and Methods. mRNA expression levels were determined by real-time RT-PCR and are expressed as each mRNA level/GAPDH. Bars represent mean ± SD, *n* = 3, Tukey–Kramer multiple comparison test. *: *p* < 0.05, **: *p* < 0.01.

Since the HC-stimulated expression of ceramide synthesis-related genes tended to be generally confined to the differentiating conditions of NHKs, we next determined the effects of agonists or antagonists of PPARs on the HC-stimulated mRNA expression levels of GCS, CERS3 and ELOVL4 only under the differentiating conditions of NHKs. Real-time RT-PCR analysis revealed that GW6471, an antagonist of PPARα, failed to abolish the HC-up-regulated mRNA expression levels of the three ceramide synthesis-related enzymes tested, GCS, CERS3 and ELOVL4, at 48 h after treatment with HC (at 200 µg/mL) (Figure 8a), whereas GSK0660, an antagonist of PPARβ/δ, significantly abrogated the HC-increased mRNA expression levels of the three ceramide synthesis-related enzymes tested, GCS, CERS3 and ELOVL4 at 48 h after treatment with HC (at 200 µg/mL) (Figure 8b). On the other hand, GW9662, an antagonist of PPARγ, significantly abolished the HC-up-regulated mRNA expression levels of GCS and ELOVL4, but not of CERS3, at 48 h after treatment with HC (at 200 µg/mL) (Figure 8c). In agreement with the above results, GW501516, an agonist of PPARβ/δ, significantly up-regulated the mRNA expression levels of GCS, CERS3 and ELOVL4 at 48 h after sham treatment (HC at 0 µg/mL) (Figure 8b). Troglitazone, an agonist of PPARγ, significantly up-regulated the mRNA expression levels of GCS and ELOVL4, but not of CERS3, at 48 h after sham treatment (HC at 0 µg/mL) (Figure 8c). On the other hand, WY14643, an agonist of PPARα, failed to stimulate the mRNA expression levels of GCS, CERS3 and ELOVL4 at 48 h after sham treatment (HC at 0 µg/mL) (Figure 8a). These results indicate that the HC-stimulated mRNA expression levels of GCS/ CERS3/ELOVL4 and GCS /ELOVL4 are predominantly mediated via PPARβ/δ- and PPARγ-associated signal activation in NHKs, respectively.

## 3. Discussion

The results of this study demonstrate for the first time that repeated treatments of cultured RHEEs with HC for 10 days significantly up-regulated the levels of SC ceramides, accompanied by significantly decreased TEWL values after 6 days of different lines of cultured RHEEs. The increased levels of SC ceramides comprise significantly increased levels of ceramide species such as Cer[EOS], Cer[NS/NDS], Cer[AS], Cer[NH] and Cer[AP], and slightly increased levels of Cer[NP]. Based on available evidence for the attribution of ceramide species from either glucosylceramide or sphingomyelin [40,41], that pattern toward increased levels of ceramide species seems to indicate that both pathways, i.e., via sphingomyelin and glucosylceramide, are stimulated by HC treatment in RHEEs.

Because the HC used in this study is a mixture of lipids containing GalCer (72~82%) as the main component along with sphingomyelin, phosphatidylethanolamine, lecithin (combined 8~25%) and cholesterol (0.1~0.6%), it is probable that the other components, not including galactosyceramide, also elicit an increase in the SC ceramide contents. Among the other components, only 1.0% sphingomyelin-based liposomes with less than 190 nm of vesicle size applied to the SC side of the same RHEEs was reported to have an incremental effect (about 120% up) on ceramide II (Cer[NDS]) and V (Cer [AS] in the same RHEEs system [42]. Thus, in our same application to the SC side of RHEEs—because, if possible, HC at 2% is estimated to contain at most 0.5% sphingomyelin suspended in 4% glycerin aqueous solution—it is not likely that such 0.5% non-liposome type of sphingomyelin contaminated may increase the SC ceramide contents in our RHEEs study, which suggests the major role of galactosylceramide in stimulating ceramide synthesis.

Ceramide levels in the SC are primarily regulated by the balance of enzymatic activities of ceramide-releasing enzymes, GBA [40,43,44,45,46] and ASM [47], and a ceramide-hydrolyzing enzyme, aCDase [48]. In the granular and stratum spinosum layers, ceramides synthesized as intermediates are converted to glucosylceramides and sphingomyelin by GCS [49,50,51] and by SMS [52,53], respectively, which accumulate in lamellar granules in the Golgi apparatus. Those granules are then secreted from the cisterna area of the Golgi apparatus and undergo exocytosis into the interface between the granular and SC layers. The intermediate ceramides are synthesized by DES [53] after dihydroceramide is produced by the action of CERS [54]. ELOVL catalyzes the first rate-limiting step of the fatty acid elongation cycle [55] and coordinately acts with CERS3 to attach long chain fatty acids to ceramides [54]. The first step of the sphingolipid-metabolizing reaction occurs with SPT [56], which catalyzes the condensation of serine and palmitoyl-CoA in epidermal basal cells to yield 3-ketosphinganine that is converted by 3-ketosphinganine reductase to sphinganine as a precursor for dihydroceramide [57,58].

According to the ceramide-related synthetic pathways as described above, we next investigated the effects of HC on the expression levels of ceramide synthesis-related genes and proteins. Quantitative RT-PCR analysis revealed that HC significantly enhanced the mRNA expression levels of CERS3, ELOVL4, GBA, GCS, SMS1 and ASM but not of SPT1, SPT2, DES1, SMS2 and aCDase. Our results that the mRNA level of GBA is up-regulated by HC treatment are in accordance with previous reports by Hara et al. in mouse skin treated with GalCer [25]. Since an increased level of the substrate glucosylceramide at least is required for up-regulated GBA activity to result in increased SC ceramide levels, it seems reasonable to assume that major synthetic linkages responsible for the HC-up-regulated level of SC ceramide are attributable to enzymes other than GBA even in the GlcCer pathway. In fact, other ceramide synthesis-related enzymes, such as CERS3, ELOVL4 and GCS, which can contribute to the increased synthesis of glucosylceramide, are also accentuated at the mRNA expression level by HC treatment.

In the present study, the increased mRNA expression levels of GCS and GBA as well as SMS1 and ASM strongly support our hypothesis that HC stimulates both pathways via sphingomyelin and glucosylceramide in RHEEs, leading to increased levels of SC ceramides. Thus, the significant increases in mRNA expression levels of SMS1 and ASM that are predominantly involved in the sphingomyelin pathway are mechanistically associated with increased levels of Cer[AS] and Cer[NS/NDS] [40,41] in the SC of RHEEs. Similarly, the significant increases in mRNA expression levels of GCS and GBA that are predominantly involved in the glucosylceramide pathway substantially contribute to increased levels of Cer[EOS], Cer[NS/NDS], Cer[AS], Cer[NH] and Cer[AP] [40,41] in the SC of RHEEs. It is also likely that the significant increases in mRNA expression levels of CERS3 and ELOVL4 that are predominantly involved in the synthesis of ceramides with a long acyl chain moiety [35] reasonably account for the increased levels of acyl-ceramide Cer[EOS] in the SC of RHEEs. Western blotting analysis of ceramide synthesis-related enzymes revealed that treatment of RHEEs with HC for 4 days significantly upregulates the protein levels of ELOVL4, CERS3, GBA, GCS and SMS1 but not of ASM. These findings indicate that, as suggested by RT-PCR analysis of ceramide synthesis-related enzymes, these significant increases in the protein levels of ELOVL4, CERS3, GBA, GCS and SMS1 are definitely attributable to the significantly increased levels of SC ceramides synthesized via both the GlcCer and SM pathways, such as Cer[EOS], Cer[NS/NDS], Cer[AS], Cer[NH], Cer[AP] and Cer[NS/NDS] and Cer[AS], respectively [40,41].

Since we have already studied the stimulatory effects of β-sitosterol 3-O-D-glucoside (BSG) [30] and tiliroside or strawberry seed extract (SSE)[29] on the SC ceramide levels in the same RHEE system, these comparisons prompted us to precisely characterize the stimulatory effects of HC in terms of the mechanisms involved in the up-regulation of ceramide synthesis-associated enzymes. Thus, BSG significantly increased the total ceramide content by 1.2-fold compared to the control in the SC of the RHEE model, accompanied by a significant increase of the ceramide species, Cer[EOS] by 2.1-fold compared to the control. This stimulatory effect on ceramide production is accompanied by significantly up-regulated mRNA expression levels of SPT2, CERS3, GCS and ASM, of 1.41–1.89, 1.35–1.44, 1.19 and 2.06-fold, respectively, compared to the non-treated control in the RHEE model. The SSE elicits a significant increase in the total ceramide content in the SC, which is accompanied by a significant increase in almost all ceramide species except for Cer[EOS] and Cer[AP]. This stimulatory effect on ceramide production is accompanied by significantly up-regulated mRNA expression levels of SPT2, CERS3, GCS and GBA. On the other hand, tiliroside slightly accentuates the total ceramide content in the SC together with a significant increase in the Cer[NS,NDS] content. This stimulatory effect on ceramide production is accompanied by significantly up-regulated mRNA expression levels of GCS and GBA only. These comparisons indicate that the stimulatory pattern of HC for ceramide synthesis-related enzymes responsible for the increased SC ceramide levels occurs similarly to that induced by SSE and that CERS3, GCS and GBA are three core ceramide synthesis-related enzymes responsible for stimulating SC ceramide levels in the RHEE system. It is most likely that the up-regulated expression levels of GBA observed in this study may contribute to an increase in the SC ceramide levels because the generation of its substrate glucosylceramide is possibly stimulated by the up-regulated level of its synthesizing enzyme, GCS.

Although the RHEE system can evaluate the effects of chemicals on all processes from basal cell proliferation to differentiation/keratinization toward the SC layer, it is difficult to determine which process is predominantly influenced by HC treatment. It is well known that high calcium conditions stimulate keratinocyte differentiation in NHKs and that mRNA expression levels of keratinization-related factors such as INV and TGM1 are markedly increased in high calcium medium conditions of NHKs in culture [59]. Therefore, as for the mechanism(s) involved in the HC up-regulated expression of ceramide synthesis- and keratinization-related genes, we elucidated the effects of HC treatment on ceramide synthesis- and keratinization-related enzymes and proteins at the mRNA level under proliferating and differentiating conditions of NHKs. Our results indicate that while mRNA expression levels of ceramide synthesis-related enzymes are generally higher in differentiating conditions than in proliferating conditions, the same enzymes, including CERS3, ELOVL4, GCS and ASM, are significantly up-regulated at the mRNA level by HC treatment only under differentiating conditions. Similarly, while the mRNA expression levels of keratinization-related genes are generally higher in differentiating conditions than in proliferating conditions, the same proteins or enzymes tested such as INV and TGM1, are significantly stimulated by HC treatment under both differentiating and proliferating conditions. Mechanistic analyses using NHKs revealed that the marked stimulatory effects of HC on the expression levels of ceramide synthesis-related genes encoding CERS3, ELOVL4 and GCS occur predominantly under high calcium-derived differentiating conditions. The different stimulatory effects of HC treatment on the mRNA expression levels of ceramide synthesis- and keratinization-related enzymes and proteins may be ascribed to different keratinization stages in their cellular locations between the enzyme and protein groups. Thus, it is likely that HC may trigger a certain convergent factor in the intracellular signaling linkages to stimulate both ceramide synthesis- and keratinization-related gene levels via unknown mechanisms.

As for the signaling mechanisms involved in HC-stimulated ceramide synthesis, it should be noted that a major component of SSE, tiliroside, which we have already reported has a stimulatory effect on SC ceramide production [29], is known to have the ability to enhance the expression of PPARα [60]. Available evidence also indicates that the activation of PPARα can stimulate the expression of ceramide-related enzymes including SPT, GBA, GCS and ASM [60]. In this connection, Mizutani et al. recently reported that the mRNA expression levels of ELOVL4 and CERS3 are up-regulated during keratinocyte keratinization in vivo and in vitro via the intracellular signaling mechanism involved in PPARβ/δ as well as PPARγ [35]. These lines of evidence for the mechanistic relationship between the expression of both ceramide synthesis- and keratinization-related proteins and enzymes and activation of PPARs support the possibility that the HC-accentuated expression of ceramide synthesis-related enzymes tested in this study, including GCS, CERS3, ELOVL4, ASM, SMS1, SMS2 and GBA, as well as of keratinization-related proteins, including CNFN, SPRR2B, TGM1, TGM3 and INV, in RHEEs and/or in NHKs may be mediated via activated signaling mechanisms involved in PPARs. Therefore, with this signaling mechanism in mind, we focused on the contribution of the activation of PPARs.

PPARs are members of the nuclear receptor family of transcription factors. There are three PPAR isoforms (PPARα, PPARβ/δ and PPARγ) in mammals, which modulate the expression levels of numerous genes involved in lipid metabolism and differentiation of keratinocytes [61]. PPARβ/δ exists as the most abundant PPAR isoform in fetal epidermis and it plays important roles in SC and cutaneous barrier formation [62,63]. In addition, levels of epidermal barrier CERs, such as w-O-acyl CER, are accentuated by the activation of PPARβ/δ in hairless mice [64,65]. On the other hand, PPARβ/δ knockout mice have a significant delay in cutaneous barrier recovery after barrier disruption [66]. Therefore, we determined whether the stimulatory effect of HC on ceramide synthesis-related enzymes at the gene level is accompanied by up-regulated mRNA expression levels of PPARs and is mediated via the activation of PPARs. Real-time RT-PCR analysis revealed that in proliferating conditions, mRNA expression levels of PPARα and PPARβ/δ, but not of PPARγ, are significantly increased by HC treatment. In differentiating conditions, mRNA expression levels of PPARβ/δ are significantly up-regulated by the treatment with HC, whereas the mRNA expression levels of PPARα and PPARγ remain unchanged. These enhanced mRNA expression levels of PPARs by HC are an important factor for accelerating signaling processes that lead to the increased expression of ceramide synthesis-related enzymes. Nevertheless, to elucidate a direct contribution of PPARs signaling to the HC-elicited up-regulation of genes encoding ceramide synthesis-related enzymes, we next determined the effects of agonists or antagonists on non-treated and HC-increased levels, respectively, of the mRNA expression levels of GCS, ELOVL4 and CERS3 under the differentiating conditions of NHKs. Analyses using agonists of PPARs under differentiating conditions revealed that GW501516, an agonist of PPARβ/δ, significantly up-regulated the mRNA expression levels of GCS, CERS3 and ELOVL4. Troglitazone, an agonist of PPARγ, also significantly enhanced the mRNA expression levels of GCS and ELOVL4, but not of CERS3. On the other hand, WY14643, an agonist of PPARα, failed to stimulate the mRNA expression levels of GCS, CERS3 and ELOVL4. This mechanistic relationship between the activation of PPARs and the expression of CERS3 and ELOVL4 is consistent with the results reported by Mizutani et al. [35] and suggests the possibility that the HC-up-regulated mRNA expression levels of GCS, CERS3 and ELOVL4 may be mainly mediated via PPARγ and/or PPARβ/δ but not PPARα. To verify that hypothesis, analyses using antagonists of PPARs under differentiating conditions demonstrated that an antagonist of PPARβ/δ significantly abrogated the HC-stimulated mRNA expression levels of GCS, CERS3 and ELOVL4, which is in agreement with the observed stimulatory effects of its agonist. GW9662, an antagonist of PPARγ, significantly abolished the HC-up-regulated mRNA expression levels of GCS and ELOVL4, but not of CERS3, which is also in agreement with the observed stimulatory effects of its agonist. These findings suggest that HC has the distinct potential to accentuate the expression of GCS, CERS3 and ELOVL4 via the activation of PPARβ/δ and/or PPARγ to accelerate ceramide synthesis in the SC.

As for an essential component of HC for up-regulation of ceramide synthesis-related enzymes and the activation of PPAR signaling, Hara et al. [25] demonstrated that galactosylceramide enhances the activity of GBA in mouse skin and in the cultured NHKs. Furthermore, Mao-Qiang et al. [59] reported that each agonist of PPARα (WY14643), PPARδ (GW1514) and PPARγ (ciglitazone) up-regulates the activity of epidermal GBA in the skin of hairless mice. These findings strongly suggest the major role of galactosylceramide as a main component of HC in stimulating SC ceramide synthesis via activated PPAR signaling lineages.

In conclusion, taken together, our results suggest that the treatment of NHKs with HC has the distinct potential to stimulate epidermal ceramide synthesis, including acyl ceramides, through maturation of the CE and the up-regulation of both the GlcCer and SM pathways by activating PPARβ/δ and/or PPARγ. Thus, HC is an excellent candidate to improve water-holding and skin barrier functions in several dry skin diseases, such as senile xerosis and AD.

## 4. Materials and Methods

### 4.1. Materials

HC was purchased from Yamakawa (Tokyo, Japan). HC extracted from horse spinal cord is a mixture of lipids containing GalCer(72~82%) as the main component along with sphingomyelin, phosphatidylethanolamine, lecithin (combined 8~25%) and cholesterol (0.1~0.6%). The GalCer of HC contains more than 11 types of molecular species. The structures with the highest content in HC are d18:14E-C24:0GalCer and d18:14E-C24h:0 GalCer.

Ceramide standards, Cer[NS/NDS], Cer[NP] and Cer[AS], used for quantitative determination, were purchased from Takasago International Corp. (Tokyo, Japan), Evonik Industries (Essen, Germany) and Matreya (State College, PA, USA). RHEEs (LabCyte EPIMODEL 6D) and assay medium were purchased from Japan Tissue Engineering (Aichi, Japan). NHKs and assay medium (Humedia-KG2) were purchased from Kurabo (Tokyo, Japan). PBS (-) was purchased from Fujifilm Wako Pure Chemical Corp. (Osaka, Japan), 0.05% Trypsin-EDTA was purchased from Gibco (BRL, Palo Alto, CA, USA) and fetal bovine serum (FBS) was obtained from Sigma-Aldrich (St. Louis, MO, USA). Isogen was purchased from Nippon Genes (Tokyo, Japan). The One-step Syber Prime Script PLUS RT-PCR kit was purchased from Takara Bio (Shiga, Japan). PCR primers were purchased from Hokkaido System Science (Hokkaido, Japan). IGEPAL™ CA-630 and cOmpl™TM) Mini Protease Inhibitor Cocktail Tablets were purchased from Sigma-Aldrich (St. Louis, MO, USA). The DC Protein Assay Kit, Laemmli sample buffer and Clarity Western ECL Substrate were from Bio-Rad Laboratories (Hercules, CA, USA). Can Get Signal solutions 1 and 2 were purchased from Toyobo (Osaka, Japan). Antibodies to ELOVL4 (DF4037) were obtained from Affinity Biosciences (Cincinnati, OH, USA). Antibodies to CERS3 (Anti-LASS3: ab169259), ASM (ab83354) and INV (ab53112) were obtained from Abcam (Cambridge, UK) and TGM3(NBP1-86950) was from Novus Biologicals (Littleton, CO, USA). Antibodies to SMS1 (bs-4216R) and GCS (bs-8593R) were obtained from Bioss (Beijing, China). Antibodies to GBA (G4171) and beta-Actin (A1978) and horseradish peroxidase (HRP) conjugated goat anti-mouse IgG and HRP conjugated goat anti-rabbit IgG were obtained from Sigma-Aldrich (St. Louis, MA, USA). Antibodies to TGM1 (12912-3-AP) were obtained from Proteintech Group (Rosemont, IL, USA). Agonists and antagonists to PPARs, WY14643/GW501516, GW6471/GSK0660 and Rosiglitazone/GW9662 were obtained from Adipogen Life Sciences (San Diego, CA, USA), Cayman Chemical (Ann Arbor, MI, USA) and Fujifilm Wako Pure Chemical Corp. (Osaka, Japan), respectively. TLC plates for HPTLC were obtained from Merck Millipore (Darmstadt, Germany). All other reagents were obtained commercially and used without further purification.

### 4.2. Preparation of HC Sample

In the experiments for real-time RT-PCR, Western blotting and HPTLC, HC was dissolved in 4% glycerin at final concentrations of 0, 10 and 20 mg/mL by homogenizing the mixture with an ultrasonic homogenizer NR-50M (Microtec, Chiba, Japan). Fine emulsions were subsequently prepared by high-pressure homogenization of the coarse emulsions using a Nano Vater NV200 (Yoshida Kikai, Nagoya, Japan). In the experiments for TEWL measurement, HC was dissolved in dimethyl sulfoxide (DMSO) and was suspended at 1 and 10 mg/mL in PBS by homogenizing the mixture with an ultrasonic homogenizer NR-50 M. For treatment of NHKs, HC was dissolved in dimethyl sulfoxide and was added to the culture at final concentration of 200 µg/mL.

### 4.3. Culture of RHEEs and Treatment with HC

RHEEs obtained prior to keratinization without development of the SC [27,28,29,30] were used to determine the expression of ceramide synthesis-related enzymes, to quantify SC ceramide levels and to evaluate TEWL. RHEEs were cultured in medium at 37 °C in a 5% CO_2_ atmosphere. To imitate the application for in vivo treatment at higher doses of HC, HC was solubilized in 4% glycerin aqueous solution at 10 or 20 mg/mL after which the HC glycerin solution was applied daily for 3 h at 50 μL/well on the surface of each RHEEs, which was then washed with PBS to remove the HC solution. The same treatment for 3 h with the HC solution and its subsequent removal were repeated daily for 10 days. The RHEEs were then subjected to analyses using real-time RT-PCR to determine gene expression levels, Western blotting to detect protein levels and HPTLC to measure SC ceramide levels at 2, 4 and 10 days of culturing. In the experiments for TEWL measurement, HC suspended at 1 and 10 mg/mL in PBS was applied daily for 3 h at 50 μL/well on the surface of each RHEEs, which was then washed with PBS to remove the HC solution. The same treatment for 3 h with the HC/ PBS solution and its subsequent removal were repeated daily for 6 days.

### 4.4. Evaluation of the Residual Level of HC Applied on RHEEs

We determined the residual levels of HC (solubilized in 4% glycerine aqueous solution at 10 or 20 mg/mL) applied for 3 h and then washed with PBS on RHEEs after culture for 1 day. After the treatment for 3 h with HC and its removal by washing with PBS, the entire tissue was peeled from the membrane and stored at −80 °C until determinations were performed. Lipid extraction was carried out using a method described in a previous report [28]. Namely, RHEEs were homogenized using an ultrasonic homogenizer NR-50M in a mixture of chloroform, methanol and PBS (1:2:0.8), and chloroform (1 mL) and PBS (1 mL) were added to each supernatant and mixed for 20 min. After mixing, the bottom layer was collected. The collected layer was dried at 30 °C using N_2_ gas. Galactosylceramide was identified and then measured by HPTLC. The HPTLC was developed twice with chloroform: methanol: acetic acid = 190:9:1 (*v*/*v*) and once with chloroform: methanol: acetone = 76:20:4 (*v*/*v*), then visualized using 10% CuSO_4_ in 8% H_3_PO_4_ solution with heating to 180 °C.

### 4.5. Culture of NHKs

NHKs were seeded at 1.6 × 10^5^ cells/well in 6-well culture plates and were maintained in Humedia-KG2 at 37 °C in a 5% CO_2_ atmosphere. After overnight incubation, the medium was exchanged with proliferation medium (Humedia-KG2, final Ca^2+^ concentration: 0.15 mM) or differentiation medium (excluding insulin, hEGF, hydrocortisone and BPE from Humedia-KG2 and final Ca^2+^ concentration: 1.5 mM), and then HC (final concentration of 200 µg/mL), agonists of PPARs (WY14643, GW501516 and troglitazone at final concentrations of 20, 20 and 5 µg/mL, respectively) or antagonists of PPARs (GW6471, GSK0660 and GW9662 at final concentrations of 4, 20 and 20 µg/mL, respectively) dissolved in 0.2% dimethyl sulfoxide were added to the medium. An amount of 0.2% dimethyl sulfoxide was added as a control. The NHKs were then cultured for 6, 12, 24 or 48 h and analyzed using real-time RT-PCR.

### 4.6. Quantification of Ceramides in the SC of RHEEs

Lipids were extracted from the SC and ceramide analysis was performed using a previously described method [28,29,30]. After culturing, the RHEEs were peeled from the membrane, and the SC was separated from epidermal cells by incubation in trypsin (2.5 mg/mL)/EDTA (0.2 mg/mL) aqueous solution at 37 °C for 1 h. The isolated SC was washed with PBS and was stored at −80 °C until used. Lipids were extracted from the isolated SC using a modification of the Bligh/Dyer method [67]. The amounts of ceramides were determined by HPTLC as described by Imokawa et al. [8]. The lipid extracts from the SC were dissolved in chloroform/methanol (2:1 by vol.) solvent and were fractionated by TLC on silica gel plates (Merck). Ceramides were developed twice over 9 cm with chloroform/methanol/acetic acid (190:9:1 by vol). The dried plates were sprayed with 10% CuSO_4_ in 8% H_3_PO_4_ solution and were charred at 180 °C. The charred plates were scanned and analyzed using ImageJ software (NIH Image, Bethesda, MD, USA). Species of ceramides were assigned according to Motta nomenclature as well as Masukawa ceramide characterization [68,69,70]. Cer[NS/NDS], Cer[NP] and Cer[AS] were used as ceramide standards for quantitative determination.

### 4.7. RNA Extraction and Real-Time RT-PCR

Whole RHEEs or NHKs were harvested in Isogen II and mRNAs were extracted according to the Isogen II protocol. Real-time RT-PCR amplification of each mRNA was performed with a Step One Plus (Life Technologies, Carlsbad, CA, USA) and One-Step SYBR Prime Script PLUS RT-PCR kit. Primers used are shown in Table 1. The mRNA expression level of GAPDH was used to correct that of each enzyme.

### 4.8. Western Blotting

Whole RHEEs were homogenized in a lysis buffer consisting of 0.1 M Tris-HCl, pH 7.4, containing 1% Igepal CA-630, 0.01% SDS and a protease inhibitor mixture. The mixture was centrifuged (4 °C, 15,000 rpm, 10 min) and the supernatant was collected. The protein content of each supernatant was adjusted to 1.0 mg/mL and was mixed with an equal volume of Laemmli sample buffer (including 5% 2-mercaptoethanol). After heating at 95 °C for 5 min, the sample solution was electrophoresed on 10% SDS-PAGE gels and the separated proteins were transferred to polyvinylidene difluoride membranes. After blocking with 5% skim milk, the membranes were treated with a primary antibody followed by a secondary antibody. Anti-ELOVL4 (1:500), anti-CerS3 (1:1000), anti-GCS (1:500), anti-SMS1(1:500), anti-GBA (1:500), anti-ASM (1:500), anti-TGM1 (1:1000) and anti-β-actin (1:2000) antibodies were used as primary antibodies. HRP-conjugated goat anti-rabbit IgG (1:2000) and HRP-conjugated goat anti-mouse IgG (1:10,000) were used as secondary antibodies. Lumicube plus (LIPONICS, Tokyo, Japan) and Clarity Western ECL Substrate were used for the detection.

### 4.9. TEWL Measurement in RHEEs

Skin barrier function was evaluated by measuring TEWL with a handy TEWL measuring instrument (VAPO SCAN AS-VT100RS, Asahi Techno Lab, Yokohama, Japan). TEWL was measured after standing RHEEs on a hot plate at 37 °C on a clean bench for 30 min [71].

### 4.10. Statistics

All results are expressed as mean ± standard deviation (SD). Comparisons between two groups were performed using Student’s *t*-test. For multiple comparisons, data were analyzed using one-way analysis of variance (ANOVA) followed by Dunnett’s test or the Tukey–Kramer multiple comparison test. A value of *p* < 0.05 was considered statistically significant.

## Figures and Tables

**Table 1:** RT-PCR primer sequences.

Primer Name		Sequence
SPT1	Forward Reverse	5′-GCGCGCTACTTGGAGAAAGA-3′ 5′-TGTTCCACCGTGACCACAAC-3′
SPT2	Forward Reverse	5′-AGCCGCCAAAGTCCTTGAG-3′ 5′-GAAGGTGAAGGTCGGAGTCAACG-3′
ELOVL4	Forward Reverse	5′-CATGTGTATCATCACTGTACG-3′ 5′-AAAGGAATTCAACTGGGCTC-3′
CERS3	Forward Reverse	5′-ACATTCCACAAGGCAACCATTG-3′ 5′-CTCTTGATTCCGCCGACTCC-3′
DES1	Forward Reverse	5′-GCGTTTGGCAGTTGCATTAA-3′ 5′-CATTGTGGGCAATCTCATGAA-3′
GCS	Forward Reverse	5′-ATGTGTCATTGCCTGGCATG-3′ 5′-CCAGGCGACTGCATAATCAAG-3′
GBA	Forward Reverse	5′-TGGCATTGCTGTACATTGG-3′ 5′-CGTTCTTCTGACTGGCAACC-3′
SMS1	Forward Reverse	5′-CAACATTGGCGTAGACAT-3′ 5′-TAGGAGGTACTCGTTCGTG-3′
SMS2	Forward Reverse	5′-ACTACTCTACCTGTGCCTGG-3′ 5′-AGCAGCCAGCAGATTAAATG-3′
ASM	Forward Reverse	5′-TGGCTCTATGAAGCGATGG-3′ 5′-AGGCCGATGTAGGTAGTTGC-3′
aCDase	Forward Reverse	5′-CCTTCTTCCTTGATGATCGC-3′ 5′-GTGGAGTTCACCATGGTTCG-3′
INV	Forward Reverse	5′-GGCCACCCAAACATAAATAACCAC-3′ 5′-CACCTAGCGGACCCGAAATAAGT-3′
HRNR	Forward Reverse	5′-AGGACAGGGCTATAGTCAGCA-3′ 5′-CCGAAGCGTGATGGGAGG-3′
LOR	Forward Reverse	5′-GAGTTGGAGGTGTTTTCCAGGG-3′ 5′-GCAGAACTAGATGCAGCCGGA-3′
CNFN	Forward Reverse	5′-ACACAGGTCTCACGGACTG-3′ 5′-CAGCACTCGC CAAAGTCGT-3′
SPRR2B	Forward Reverse	5′-GCCTGGGAACCATCAGAGAA-3′ 5′-TCATGCCCAGGTGAAAGACA-3′
TGM1	Forward Reverse	5′-GCTCGAAGGCTCTGGGTTACA-3′ 5′-TCGCACAGGCACAAACGAC-3′
TGM3	Forward Reverse	5′-TCACACAGACAAGTTCTCCA-3′ 5′-ATTGGAGAGTGGAAACACAG-3′
PPARα	Forward Reverse	5′-GGTGGACACGGAAAGCCCAC-3′ 5′-GGACCACAGGATAAGTCACC-3′
PPARβ/δ	Forward Reverse	5′-CACACGGCGCCCTTTG-3′ 5′-CCTTCTCTGCCTGCCACAA-3′
PPARγ	Forward Reverse	5′-ATTCTGGCCCACCAACTTTG-3′ 5′-TCCATTACGGAGAGATCCACG-3′
GAPDH	Forward Reverse	5′-GAAGGTGAAGGTCGGAGTCAACG-3′ 5′-AGTCCTTCCACGATAACCAAAGTTG-3′

## Data Availability

Not applicable.

## Abbreviations

Acid ceramidase (aCDase), acid sphingomyelinase (ASM), acid-sphingomyelinase (ASM), atopic dermatitis (AD), ceramide synthase (CERS3), coniferin(CNFN), cornified envelope (CE), dihydroceramide desaturase (DES), fatty acid elongases (ELOVL), galactosylceramide (GalCer), glucosylceramide synthase (GCS), Glyceraldehyde-3-phosphate dehydrogenase (GAPDH), horse-derived ceramide (HC), high-performance thin-layer chromatography (HPTLC), hornerin (HRNR), horseradish peroxidase (HRP), involucrin (INV), loricrin (LOR), normal human keratinocytes (NHKs), phosphate buffer saline (PBS), peroxisome proliferator activated receptors (PPAR), reconstructed human epidermal equivalents (RHEEs), serine palmitoyltransferase (SPT), small proline-rich protein (SPRR), sphingomyelin synthase (SMS), standard deviation (SD), stratum corneum (SC), strawberry seed extract (SSE), transglutaminase (TGM), β-Sitosterol 3-O-D-glucoside (BSG), β-glucocerebrosidase (GBA), trans-epidermal water loss (TEWL).
